# Cyclin E and CDK2 Repress the Terminal Differentiation of Quiescent Cells after Asymmetric Division in *C. elegans*

**DOI:** 10.1371/journal.pone.0000407

**Published:** 2007-05-02

**Authors:** Masaki Fujita, Hisako Takeshita, Hitoshi Sawa

**Affiliations:** 1 Laboratory for Cell Fate Decision, RIKEN, Center for Developmental Biology, Kobe, Japan; 2 Department of Biology, Graduate School of Science, Kobe University, Kobe, Japan; Ecole Normale Superieure, France

## Abstract

Coordination between cell proliferation and differentiation is important in normal development and oncogenesis. These processes usually have an antagonistic relationship, in that differentiation is blocked in proliferative cells, and terminally differentiated cells do not divide. In some instances, cyclins, cyclin-dependent kinases (CDKs) and their inhibitors (CKIs) play important roles in this antagonistic regulation. However, it is unknown whether CKIs and cyclin/CDKs regulate the uncommitted state in quiescent cells where CDK activities are likely to be low. Here, we show in *C. elegans* that *cye-1*/cyclin E and *cdk-2*/CDK2 repress terminal differentiation in quiescent cells. In *cye-1* mutants and *cdk-2(RNAi)* animals, after asymmetric division, certain quiescent cells adopted their sister cells' phenotype and differentiated at some frequency. In contrast, in *cki-1(RNAi)* animals, these cells underwent extra divisions, while, in *cki-1(RNAi); cdk-2(RNAi)* or *cki-1(RNAi); cye-1* animals, they remained quiescent or differentiated. Therefore, in wild-type animals, CKI-1/CKI in these cells maintained quiescence by inhibiting CYE-1/CDK-2, while sufficient CYE-1/CDK-2 remained to repress the terminal differentiation. The difference between sister cells is regulated by the Wnt/MAP kinase pathway, which causes asymmetric expression of CYE-1 and CKI-1. Our results suggest that the balance between the levels of CKI and cyclin E determines three distinct cell states: terminally differentiated, quiescent and uncommitted, and proliferating.

## Introduction

In animal development, cell proliferation and terminal differentiation must be strictly linked and coordinated [1], [2]. A disruption of this process can cause developmental abnormalities or cancer. Cyclins, CDKs, and CKIs, are key regulators of this coordination. For example, p27^xic1^/CKI is highly expressed in the terminally differentiated cells of the retina [3], and its forced expression in the retina not only blocks cell proliferation but also induces differentiation. In myoblasts, p57^kip2^/CKI induces muscle differentiation by inhibiting the cyclin E/CDK2 complex that phosphorylates and destabilizes MyoD [4]. In sensory hair cells, p19^ink4d^/CKI is required to maintain the differentiated state [5]. These observations indicate that CKI and cyclin/CDK complexes regulate not only proliferation but also an antagonistic coordination between proliferation and differentiation. Although this antagonistic relationship occurs frequently during development, some cells do not follow this rule. For example, certain stem cells have both quiescent and uncommitted characteristics and do not divide until they receive the appropriate signals [6]. In hematopoietic stem cells, p21^cip^/CKI can maintain the quiescent state [7]. However, it is not known whether CKIs and cyclins also regulate the uncommitted state of stem cells.

Even in *C. elegans*, cell-cycle regulators are involved in cell-fate acquisition. It was reported that *cki-1*/CKI (*RNAi*) animals have extra distal tip cells (DTCs), which are terminally differentiated cells that migrate and guide the gonadal arms [8]. Laser ablation and lineage analyses showed that the extra DTCs often result from extra divisions of cells that normally do not produce DTCs, indicating that *cki-1* has an important role in linking cell division with cell fate. Similarly, in *cye-1*/cyclin E mutants, some non-vulval cells adopt vulval fates [9]. However, it remains to be elucidated whether these cell-cycle regulators are involved in cell-fate acquisition directly or through their conventional functions in cell-cycle regulation. A recent report showed that a *cyd-1*/cyclin D mutant lacks DTCs [10]. In this case, an abnormal asymmetric distribution of POP-1/TCF between daughter cells indicated that *cyd-1* regulates the polarity of the first asymmetric divisions of DTC ancestors (Z1/Z4 cells). As a consequence of the abnormal polarity, both daughter cells acquire non-DTC fates in *cyd-1* mutants. A similar role in the regulation of cell polarity was reported for cyclin E in *Drosophila* [11]. These results indicate that cyclins play crucial roles in the fate determination of proliferating cells. However, it has not been shown in any organism whether cyclins and CDKs also regulate cell fate in quiescent cells.

We found that *cye-1*/cyclin E mutants in *C. elegans* have extra DTCs. By laser ablation and lineage analyses, we showed that in *cye-1* animals, the sister cells of DTCs, which are normally quiescent, differentiate into DTCs. Unlike in *cki-1(RNAi)* animals, these cells in *cye-1* mutants became DTCs within a few hours after they were born, without further cell divisions, indicating that, in normal animals, *cye-1* represses their differentiation before it functions to promote S-phase entry. We observed a similar extra-DTC phenotype in animals of *cdk-2(RNAi)*, a putative orthologue of CDK2. Our results indicate that cyclin E/CDK2 can suppress differentiation even in quiescent cells.

## Results

### The sister cells of DTCs become DTCs in *cye-1* mutants

**Figure 1:**
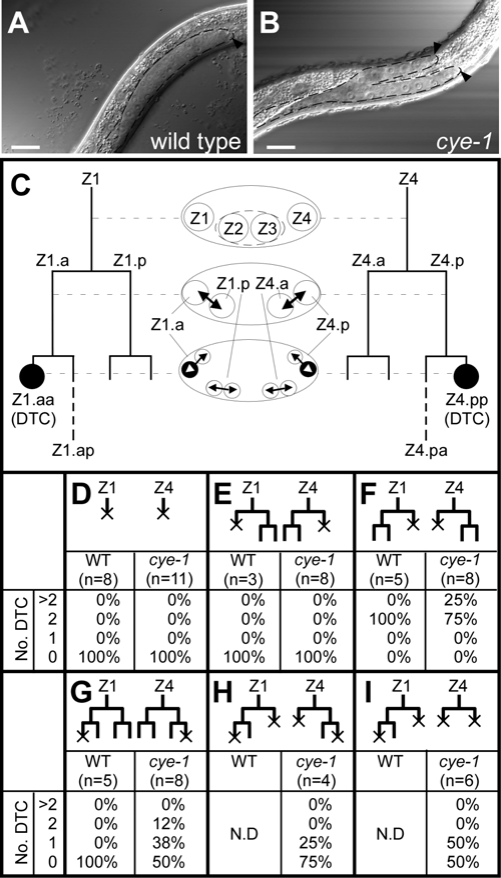
Generation of extra DTCs from the sister cells of DTCs in *cye-1* mutants. (A and B) Structure of gonads in a wild-type animal (A) and *cye-1(eh10)* mutant (B) at the L3 stage. The DTCs are marked by arrowheads. The gonad is outlined with dotted lines. Anterior is to the left; ventral is to the bottom. Anterior gonads were out of focus. Scale bar, 20 µm. (C) The lineages of the Z1 and Z4 cells during the L1 stage in wild-type animals are indicated on the left and right sides. A schematic drawing of the gonad with the positions and division axes of somatic gonadal cells is shown in the center. The Z2 and Z3 cells are primordial germ cells. The DTCs are indicated by black circles. (D–I) Laser ablation experiments in wild-type animals and *cye-1(os66)* mutants. Lineage diagrams with the ablated cells marked by an X are shown in the upper part of each panel. The lower parts of each panel show the percentages of animals that had the numbers of DTCs indicated on the left. Panel I includes animals in which Z1.a, Z1.p, Z4.a, and Z4.pp were ablated.

We found that *cye-1* mutants have extra gonadal arms (Fig. 1B; Table 1). The somatic gonad is produced from two precursor cells, Z1 and Z4 (Fig. 1C) [12]. Each of these divides to generate four cells at the L1 stage. Among the progeny, the most distal cells, Z1.aa and Z4.pp, become DTCs, which normally migrate to generate two gonadal arms, without further divisions. We found that *cye-1* mutants (*os66, ar95, eh10* and RNAi) had up to two extra DTCs per animal, all of which were positioned at the distal ends of the gonadal arms, as judged by the expression of *lag-2::GFP*, which is expressed in DTCs (Table 1) [13]. This phenotype has not been reported, even in analyses of the *cye-1(RNAi)* gonadal phenotype [10], probably because the feeding-RNAi method produces a weaker effect than we observed using RNAi injection or nonsense mutants. However, it was reported that *cye-1* mutants have an abnormally shaped gonad [9]. Such an abnormality might be caused by extra DTCs. We analyzed the divisions of the gonadal precursor cells at the L1 stage in *cye-1* mutants and found that they divided twice with the same timing and orientation as in wild type (*n*>10, data not shown).

**Table 1:** Production of extra DTCs in mutants of cell-cycle regulators.

genotype	% of extra gonad (n)		% of extra DTC (n)		P-values
wild type	0	(50)	0	(100)	–
cye-1(os66)	30	(50)	32	(106)	<0.0001^a^
cye-1(eh10)	36	(50)	36	(102)	<0.0001^a^
dpy-5(e61) cye-1(ar95)	21	(50)	56	(74)	<0.0001^a^
cye-1(RNAi)	N. D.		24	(37)	<0.0001^a^
cye-1(os66); CYE-1::GFP	N. D.		0	(20)	0.0009^b^
cdk-2(RNAi)	N. D.		20	(40)	<0.0001^a^
cye-1(os66); cdk-2(RNAi)	N. D.		25	(12)	0.445^b^
cki-1(RNAi)	N. D.		51	(41)	<0.0001^a^
cye-1(os66); cki-1(RNAi)	N. D.		17	(24)	0.1027^b^
cdk-2(RNAi); cki-1(RNAi)	N. D.		4	(84)	0.0049^c^
wild type with heat shock at middle L1	N. D.		0	(200)	–
hs::cki-1 with heat shock at early L1	N. D.		0	(56)	–
hs::cki-1 with heat shock at middle L1	N. D.		3	(196)	0.0141^d^
hs::cki-1 with heat shock at late L1	N. D.		0	(107)	–

The extra-gonad phenotype was scored under Nomarski optics. The extra-DTC phonotype was scored in strains carrying *lag-2::GFP*. In *dpy-5 ar95* animals, the extra-gonad phenotype was observed less frequently than the extra-DTC phenotype, because the shape of the gonads was difficult to observe in the *dpy-5* background, especially for gonads on the far side of the animal from the objective lens. Heat shock was applied at the early (0–5 hrs after hatching), middle (7–11 hrs after hatching), or late (13–15 hrs after hatching) L1 stage for 2 hrs at 33°C. The percentage of animals with extra gonads or DTCs is shown. n: number of animals scored. N. D.; not determined. ^a^ Compared with wild type. ^b^ Compared with *cye-1(os66)* mutants. ^c^ Compared with *cdk-2(RNAi)* animals. ^d^ Compared with wild type with heat shock at middle L1. See text.

To identify the cells that generated the extra DTCs in *cye-1* mutants, we performed laser ablation experiments (Figs. 1D–I). Ablating both the Z1 and Z4 cells yielded no DTCs in *cye-1* mutants (Fig. 1D), indicating that the extra DTCs were generated from the Z1/Z4 lineages. We next ablated the Z1 and Z4 daughter cells. When both the Z1.a and Z4.p cells were ablated in *cye-1* mutants, no DTCs were observed (Fig. 1E). In contrast, when both the Z1.p and Z4.a cells were ablated, extra DTCs were observed (Fig. 1F), indicating that the extra DTCs were generated only from the Z1.a and Z4.p cells. We next ablated both of the original DTCs (Z1.aa/Z4.pp) and found that 4/8 *cye-1* mutants still had DTCs (Fig. 1G), indicating that the extra DTCs were generated from the sister cells of the DTCs (Z1.ap/Z4.pa). To confirm these results, we ablated all the granddaughters of the Z cells except Z1.ap and Z4.pa (Fig. 1H) or except Z1.ap alone (Fig. 1I). We found that in both cases, *cye-1* mutants produced DTCs, confirming that the extra DTCs are generated from the sister cells of the DTCs.

**Figure 2:**
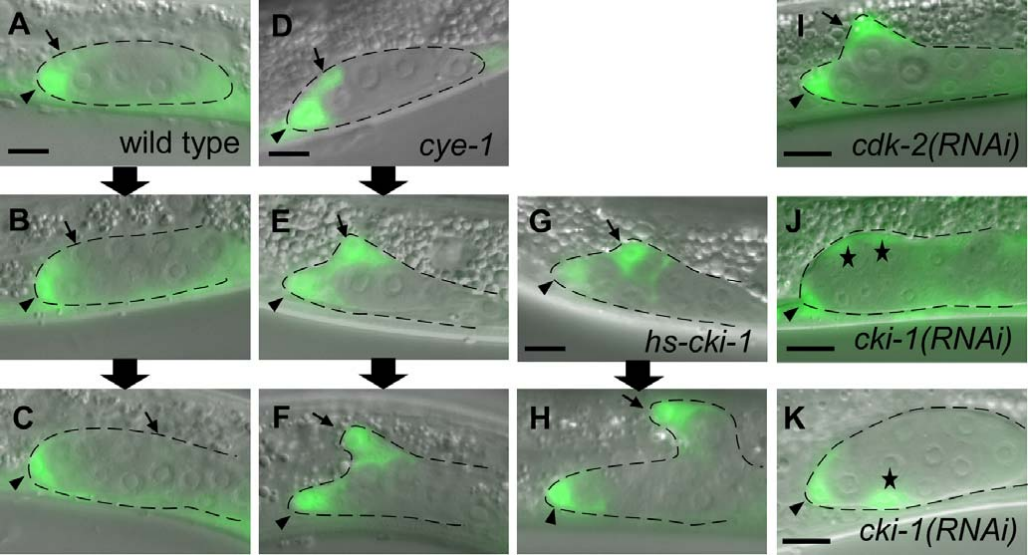
Transformation of quiescent cells to DTCs after their divisions in *cye-1* mutants. (A–K) Anterior is to the left; ventral is to the bottom. Merged GFP and Nomarski images. The gonad is outlined with dotted lines. The original DTC (Z1.aa) and its sister cell (Z1.ap) are marked by an arrowhead and arrow, respectively. The extra *lag-2::GFP*-positive cells produced from Z1.ap (J) or Z1.p (K) are indicated by asterisks. Scale bar, 10 µm. (A–H) Real-time analyses of *lag-2::GFP* expression in wild type (A–C), *cye-1(os66)* mutants (D–F), and *hs::cki-1* animals after heat shock (G and H) from the late L1 to early L2 stage. Each vertical set of panels represents the same animal over time. The expression about 3 hours (A and D), 5 hours (B, E and G), and 8 hours (C, F and H) after division of the Z1.a cell is shown. (I–K) *lag-2::GFP* expression in *cdk-2(RNAi)* (I) and *cki-1(RNAi)* animals (J and K).

We next followed the fate of the Z1.ap and Z4.pa cells in live wild-type or *cye-1* animals expressing *lag-2::GFP* (Figs. 2A–C, 2D–F). Just after the Z1.a and Z4.p cells divided, a weak GFP signal was detected in all of their daughter cells in both *cye-1* mutants and wild-type animals (*n* = 8, data not shown). In wild-type animals, after 2 to 3 hours, the GFP signal increased in the DTCs (Z1.aa/Z4.pp) and decreased in their sister cells (Z1.ap/Z4.pa) (Fig. 2A). After about 5 hours, when the DTCs started to migrate, *lag-2::GFP* was expressed nearly exclusively in the DTCs and not in their sisters (Fig. 2B). In contrast, in 3/8 *cye-1* mutants, the GFP signal increased in both daughters of Z1.a or Z4.p, 3 hours after they were born (Fig. 2D). After 5 hours, both daughter cells started to migrate like wild-type DTCs (Fig. 2E). They continued to migrate without further cell divisions for at least 8 hours after they were born (Fig. 2F). We also periodically followed the *lag-2::GFP* expression in *cye-1* mutants from the L2 to L4 stages and found that the number of DTCs did not change. These results indicate that in *cye-1* mutants, the Z1.ap/Z4.pa cells became DTCs within a few hours after they were born at the L1 stage, like the Z1.aa/Z4.pp cells.

In other organisms, cyclin E functions with CDK2. In *C. elegans*, CDK-2/K03E5.3 is the most likely orthologue of CDK2, based on sequence similarity [14]. Consistent with this possibility, both *cye-1* mutants and *cdk-2(RNAi)* animals show a protruding vulva (Pvl) and sterility [9], [14]. We found that *cdk-2(RNAi)* animals had the extra DTC phenotype (Fig. 2I; Table 1). As in *cye-1* mutants, the extra *lag-2::GFP*-positive cells in *cdk-2(RNAi)* animals were always observed at the position of Z1.ap/Z4.pa at the end of the L1 stage, and these cells migrated distally without further divisions (n = 8), indicating that the Z1.ap/Z4.pa cells had transformed into DTCs. Furthermore, this phenotype was not significantly enhanced in *cye-1; cdk-2(RNAi)* double mutants (Table 1). These results strongly suggest that CDK-2 is a partner of CYE-1.

### The Wnt/MAPK pathway regulates asymmetric expression of *cye-1* and *cki-1*

To understand how *cye-1* regulates the fates of these cells, we analyzed the expression of *cye-1* and its putative negative-regulator, *cki-1*. CKI-1 can bind CYE-1 in vitro and has been suggested to act downstream of *cyd-1/cdk-4* in cell-cycle regulation?[15]. We generated CYE-1::GFP by inserting the *gfp* gene at the C-terminus-encoding end of a *cye-1* genomic fragment that included the promoter region. CYE-1::GFP rescued the extra-DTC phenotype of *cye-1* mutants (Table 1).

**Figure 3:**
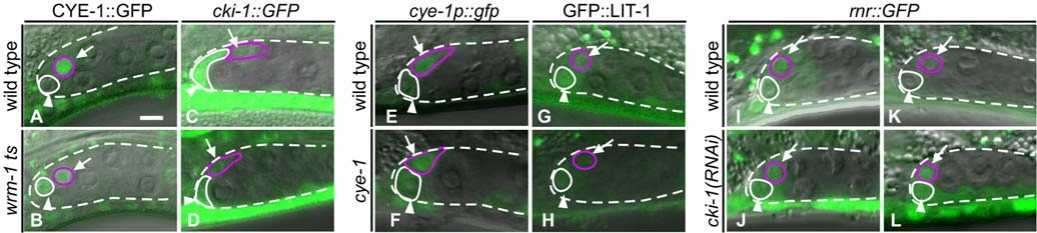
Expression of *cye-1, cki-1, lit-1* and *rnr::GFP* in the Z1.a daughters at the late L1 stage. (A–L) Anterior is to the left; ventral is to the bottom. Merged GFP and Nomarski images. The gonad is outlined with dotted lines. Scale bar, 10 µm. The nucleus (A, B, G, H and I–L) or cell membrane (C–F) of Z1.aa (arrowhead) and Z1.ap (arrow) is outlined by white and purple lines, respectively. The expression of CYE-1::GFP in wild type (A) and *wrm-1(ne1982)* mutants (B). CYE-1::GFP containing the full-length CYE-1 sequence was localized mainly to the nucleus. Expression of *cki-1::GFP* in wild type (C) and *wrm-1(ne1982)* mutants (D). *cki-1::GFP* does not include the *cki-1* coding sequence [16] and was expressed in the cytoplasm and nucleus. Expression of *cye-1* promoter::GFP (*cye-1p::gfp)* in wild type (E) and *cye-1(os66)* mutants (F). Expression of GFP::LIT-1 in wild type (G) and *cye-1(os66)* mutants (H). Expression of *rnr::GFP* in wild type (I and K) and *cki-1(RNAi)* animals (J and L). GFP was detected just after the division of Z1.a (I and J) and disappeared after 2hr in wild type (K), but not in *cki-1(RNAi)* animals (L).

CYE-1::GFP was expressed in the Z1.a/Z4.p cells before their division (data not shown). Within 2 hrs after their division, the GFP signal decreased in the Z1.aa/Z4.pp but not in the Z1.ap/Z4.pa cells (Fig. 3A). Therefore, CYE-1 is expressed asymmetrically between the daughters of the Z1.a/Z4.p cells. Similar asymmetric expression was also detected using a *cye-1* promoter*::GFP* fusion gene (*cye-1p::gfp*), which lacks the *cye-1* coding sequence (Fig. 3E), indicating that the asymmetry is regulated at the transcriptional level. In contrast, *cki-1* expression, detected by a *cki-1* promoter*::GFP* fusion gene [16], was much higher in the Z1.aa/Z4.pp cells than in the Z1.ap/Z4.pa cells (Fig. 3C). These results suggest that the asymmetric expression of *cki-1* and *cye-1* determines the different fates of the DTCs (Z1.aa/Z4.pp) and their sister cells (Z1.ap/Z4.pa).

In *C. elegans*, the asymmetry of many cell divisions is regulated by the Wnt/MAPK pathway [17]–[19]. Wnt/MAPK signaling also regulates the asymmetric nuclear localization of POP-1/TCF, LIT-1/MAP kinase, and WRM-1/ß-catenin between daughter cells [18]–[21]. A recent report showed that a mutation of *cyd-1*/cyclin D disrupts the polarity of the Z1/Z4 division, resulting in symmetric POP-1 localization [10]. The effect of this *cyd-1* mutation on the Z1.a/Z4.p divisions was not reported. To investigate the possibility that the *cye-1* mutation disrupts the polarity of Z1.a/Z4.p cells, we examined the localization of GFP::LIT-1. (We could not examine the expression of GFP::POP-1 and WRM-1::GFP, because their expression in *cye-1* mutants caused abnormal gonadal cell divisions.) GFP::LIT-1 was higher in the Z1.aa/Z4.pp than in the Z1.ap/Z4.pa cells in wild type (8/9 animals) and in *cye-1* mutants (12/13 animals) (Figs. 3G and H), suggesting that the *cye-1* mutation does not affect the polarity of the Z1.a/Z4.p cells.

We next examined whether the asymmetric expression levels of *cye-1* and *cki-1* were regulated by the Wnt/MAPK pathway, using a temperature-sensitive *wrm-1*/ß-catenin mutation (*ne1982*) [22]. To avoid disrupting the Z1/Z4 polarity in *wrm-1* mutants, the mutants were grown at the permissive temperature (15°C), and then shifted to the restrictive temperature (25°C) soon after the division of Z1/Z4. After the temperature shift, CYE-1::GFP was expressed strongly in both daughters of Z1.a/Z4.p (9/9 animals, Fig. 3B), and *cki-1::GFP* was expressed weakly in both daughters (14/15 animals, Fig. 3D), like the expression patterns in the Z1.ap/Z4.pa cells in wild-type animals. Consistent with this, the shifted animals were defective in DTC production (no DTCs in 2/20 animals and one DTC in 2/20 animals. The *P*-value was 0.0021 compared with wild type by Fisher's exact test). These results indicate that the asymmetric expression of *cye-1* and *cki-1* is regulated by the Wnt/MAPK pathway. In *cye-1* mutants, the asymmetric expression of *cye-1p::gfp* between the Z1.aa/Z4.pp and Z1.ap/Z4.pa cells was maintained (10/10 animals, Fig. 3F), suggesting that the *cye-1* mutation does not affect the initial asymmetry between the Z1.aa/Z4.pp and Z1.ap/Z4.pa cells that is generated by the Wnt/MAPK pathway.

### *cki*-1 inhibits *cye*-1 and *cdk*-2

In contrast to the Z1.aa/Z4.pp cells, which are terminally differentiated, their sisters, Z1.ap/Z4.pa, are quiescent in wild type, because they are born at the L1 stage but do not divide until the L3 stage [12]. In addition, Z1.ap/Z4.pa and Z1.aa/Z4.pp undergo extra divisions in *cki-1(RNAi)* animals (66%; n = 29) [8], indicating that *cki-1* is required for the maintenance of the quiescent state of these cells. We found that the extra divisions of these cells in *cki-1(RNAi)* animals occurred 2-5 hrs after the cells were born. We analyzed the expression of GFP driven by the *rnr* (ribonucleotide reductase) promoter, *rnr::GFP*, a marker that starts to be expressed at the S phase (Figs. 3I–L) [16]. In wild-type animals, the GFP protein remained in the Z1.a/Z4.p cells after they divided (Fig. 3I). In the Z1.ap/Z4.pa cells, it disappeared within 2 hrs after the cells were born (Fig. 3K) and reappeared at the end of the L2 stage (n = 10; data not shown), suggesting that the S phase starts at the end of the L2 stage. In *cki-1(RNAi)* animals, it was continuously expressed in the Z1.ap/Z4.pa cells during the L1 stage (63%; n = 24, Fig. 3L). These results suggest that *cki-1* starts functioning to maintain the quiescent state of these cells within 2 hrs after they are born. In *cye-1* mutants, the *lag-2::GFP* signal in these cells increased with similar timing (within about 3 hrs after they were born). Together these findings indicate that in wild-type animals, *cye-1* represses the differentiation of Z1.ap/Z4.pa cells into DTCs at the same time that *cki-1* functions to block extra cell divisions, and long before *cye-1* starts functioning to promote S-phase entry.

We next examined whether *cki-1* acts through *cye-1* and *cdk-2* to maintain the quiescent state. We scored the numbers of somatic gonadal cells derived from either the Z1.a or Z4.p cells, based on their positions and expression of *lag-2::GFP* at the early to middle L2 stage (these non-DTC cells have residual fluorescence detectable under the confocal microscope, while the fluorescence in germ cells is undetectable). At least 7/29 of the *cki-1(RNAi)* animals showed extra divisions in the Z1.a/Z4.p lineages. An additional 12/29 of these animals showed extra cell divisions from either the Z1.a/Z4.p or the Z1.p/Z4.a lineages. In contrast, in *cye-1(os66); cki-1(RNAi)* (n = 24) and *cdk-2(RNAi); cki-1(RNAi)* (n = 15) double mutants, no extra divisions were observed in the Z1.a/Z4.p lineages, even when Z1.ap/Z1.pa did not differentiate into DTCs. Therefore, *cye-1* and *cdk-2* are likely to regulate cell division as well as differentiation in the Z1.ap/Z4.pa cells. In wild-type animals, *cki-1* probably inhibits the proliferation of these cells by repressing the CYE-1/CDK-2 activities; however, sufficient CYE-1/CDK-2 function remains to repress terminal differentiation.

It was reported that *cki-1(RNAi)* animals also produce extra DTCs [8]. The lineage analyses in that report showed that the extra DTC production in the Z1.a/Z4.p lineages always occurred after extra divisions. Consistent with this finding, in all *cki-1(RNAi)* animals in which we observed *lag-2::GFP*-strong-positive cells at the positions of Z1.ap/Z4.pa (n = 5), more than two *lag-2::GFP*-strong-positive cells were observed (Fig. 2J). In contrast, in *cye-1(os66)* (n = 10), *cdk-2(RNAi)* (n = 10), *cye-1; cki-1(RNAi)* (n = 4), or *cdk-2(RNAi); cki-1(RNAi)* (n = 3) mutants, only a single *lag-2::GFP*-strong-positive cell was observed at each Z1.ap/Z4.pa position (Figs. 2E and I; data not shown). In addition to the extra DTCs derived from the Z1.a/Z4.p lineages, *cki-1(RNAi)* animals also produce extra DTCs from the Z1.p/Z4.a lineages [8]. Consistent with this, we observed extra *lag-2::GFP*-strong-positive cells at the position of Z1.p/Z4.a-derived cells (the ventral center of the gonad) in *cki-1(RNAi)* animals (Fig. 2K). In contrast, extra positive cells at this position were never observed in *cye-1, cdk-2(RNAi), cye-1; cki-1(RNAi)*, or *cdk-2(RNAi); cki-1(RNAi)* animals. These results indicate that the causes of the extra DTCs in *cki-1(RNAi)* and *cye-1/cdk-2(RNAi)* animals are different and that *cye-1* and *cdk-2* are epistatic to *cki-1* for these phenotypes.

**Figure 4.**
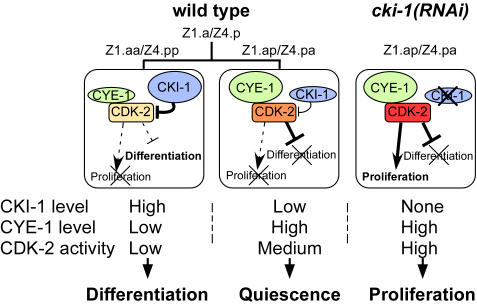
A model for the function of CKI-1 and CYE-1/CDK-2. In the Z1.aa/Z4.pp cells, highly expressed *cki-1* strongly represses the low CYE-1/CDK-2 activity, blocking proliferation and permitting differentiation into DTCs. In the Z1.ap/Z4.pa cells, the low level of CKI-1 blocks cell division by inhibiting the CYE-1/CDK-2 complex, but sufficient CYE-1/CDK-2 remains to repress terminal differentiation. In *cki-1(RNAi)* animals, a high level of CYE-1 drives the cells towards proliferation.

Our results indicate a model in which high CKI-1 and low CYE-1 levels in Z1.aa/Z4.pp result in their differentiation into DTCs, while low CKI-1 and high CYE-1 levels in Z1.ap/Z4.pa result in the quiescent state (Fig. 4, see Discussion for details). To confirm this model, we altered the balance between CKI-1 and CYE-1 by over-expressing CYE-1 using a heat-shock promoter [23] before the division of Z1.a/Z4.p, and found that the animals often lacked DTCs (18% one DTC and 27% no DTCs; n = 22). We also found that over-expression of CKI-1 using a heat-shock promoter (*hs::cki-1*) [24] resulted in extra DTCs, albeit at a low frequency (Table 1). The extra-DTC phenotype was observed only when heat shock was applied at the middle L1 stage (7 to 13 hours after hatching), which corresponds to the time just before Z1/Z4 division to soon after Z1.a/Z4.p division. As in *cye-1* and *cdk-2(RNAi)* animals, in *hs::cki-1* animals, a single, extra *lag-2::GFP*-positive cell was always observed at the position of either Z1.ap or Z4.pa at the end of the L1 stage, and this cell migrated distally during the L2 stage, indicating the transformation of the Z1.ap/Z4.pa cells into DTCs (Figs. 2G and H). The weak effect of the over-expressed *cki-1* was probably due to the high level of CYE-1 in the Z1.ap/Z4.pa cells. These results suggest that CKI-1 inhibits the activity of CYE-1/CDK-2 not only in proliferation but also in the repression of differentiation.

### *cye-1* represses the syncytial fate in quiescent seam cells

**Figure 5:**
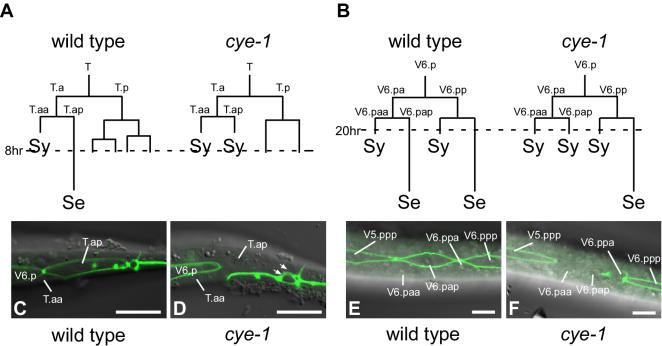
cye-1 represses the syncytial fate in seam cells. (A and B) Lineages of the T (A) and V6.p (B) cells in wild-type and *cye-1(os66)* mutants. Sy: syncytial cell. Se: seam cells. Dotted lines indicate the time after hatching the phenotype was scored (8 hrs for T and 20 hrs for V6.p). (C–F) Confocal images of AJM-1::GFP expression. T.ap and V6.pap were outlined by the fluorescence in wild-type animals (C and E) but not in *cye-1(os66)* mutants (D and F). The lack of AJM-1::GFP signal indicates that these cells fused with the hypodermal syncytium. In *cye-1* mutants, daughters of the T.p cell (arrows in D) often did not divide further. Scale bar, 10 µm.

To investigate whether *cye-1* regulates cell fates in other cell lineages, we analyzed seam cells. In *C. elegans, cye-1* adult animals have fewer seam cells than in wild type, although their differentiation is normal [9]. Seam cells are specialized hypodermal cells aligned on the lateral sides of the animal. At the end of the L4 stage, seam cells differentiate by fusing with each other and producing cuticular structures, termed alae [25]. At the early larval stages, most seam cells, including T.a, V6.pa, and V6.pp, undergo asymmetric divisions producing posterior seam cell (Se) daughters and anterior daughters, which are terminally differentiated cells that fuse with the hypodermal syncytium (Sy) within a few hours after they are born (Figs. 5A and B) [25]. Seam and syncytial cells can be distinguished by the adherence junction marker AJM-1::GFP [26], [27], which outlines seam but not syncytial cells (Figs. 5C and E) [28], [29]. We found that, in *cye-1* mutants, some of the posterior daughters of the seam cells abnormally adopted syncytial fates in the early larval stages (Figs. 5D and F; data not shown), consistent with the observation that adult *cye-1* animals have fewer seam cells than normal [9]. We scored this defect in the T and V6.p lineages, in which the penetrance appeared to be higher than for other seam cells. In *cye-1(os66)* mutants, the posterior daughters of T.a and V6.pa, which are seam cells in wild-type animals, often fused to the syncytium (9/21 for T.ap and 4/10 for V6.pap), like their sisters (T.aa and V6.paa). Because the defects were observed shortly after these cells were born, the defects were unlikely to be the indirect consequences of an abnormal cell cycle. Consistent with this, blocking the S phase by hydroxyurea soon after the T.ap cell was born did not transform it into syncytium (n = 8, data not shown). Similar to the Z1.ap/Z4.pa cells, both the T.ap and V6.pap cells appeared to be quiescent, because they did not divide until the next larval stages, and did not express the S-phase marker (*rnr::gfp)* until about 9 hrs for T.ap and 5 hrs for V6.pap after they were born (see Materials and methods; data not shown). Thus, *cye-1* appears to repress terminal differentiation in multiple quiescent cells in *C. elegans*. However, *cye-1* may not have this function in all quiescent cells, because the number of anchor cells produced from Z1.ppp/Z4.aaa after the long quiescent periods was not affected in *cye-1* mutants (n = 31), as judged by the expression of *zmp-1::GFP*, which is a marker for the anchor cell [30].

## Discussion

### Cyclin E and cell proliferation

In many organisms, progression of the cell cycle from the G1 to the S phase is controlled by the activities of CDKs and their partners, cyclins. E-type cyclins are G1 cyclins and have been thought to be required for the transition from the G1 to the S phase [31]. However, they are dispensable for normal mitotic cell division in the mouse, given that mice deficient in both cyclin E1 and E2 develop almost normally [32]. Similarly, in *C. elegans, cye-1* null homozygotes from heterozygote mothers do not show embryonic or larval lethality [9]. Even though they have variable cell-cycle defects in some lineages, like vulval cells [9], the M lineage [33], and the posterior granddaughters of the T cell (Fig. 5D), the cell divisions are not completely blocked, even in those lineages. In contrast, we showed that ectopic cell divisions in *cki-1(RNAi)* animals were completely suppressed in *cye-1* mutants, at least in the somatic gonad. Similarly, ectopic cell divisions of vulval precursor cells induced in mutants deficient in *cdc-14*, a putative regulator of *cki-1*, are also reported to be completely suppressed in *cye-1* mutants [34]. Although it is not clear whether *cye-1* is dispensable for most cell divisions because *cye-1*-null mutants may still carry maternally supplied *cye-1* products, these observations suggest that there are fundamental differences between normal and ectopically induced cell divisions in terms of their dependence on *cye-1*. In mouse, even though cyclin E-deficient cells can proliferate, they are resistant to oncogenic transformation [32]. Therefore, the roles of cyclin E may be more important for aberrant cell divisions than for divisions in normal development in both species.

### *cye-1* represses terminal differentiation in quiescent cells

We showed that *cye-1* and *cdk-2(RNAi)* animals have extra DTCs as a result of the transformation of Z1.ap/Z4.pa into their sister cells, indicating defects in asymmetric cell division. However, the polarity of the Z1.a/Z4.p divisions appeared to be normal, because the expression of GFP::LIT-1 and *cye-1p::gfp* was asymmetric between the daughters in *cye-1* mutants, as in wild type. We also showed that the Wnt/MAPK pathway regulates the asymmetric expression of CKI-1 and CYE-1 between daughter cells. In contrast, in the division of the Z1/Z4 cells, the *cyd-1* mutation affects the Wnt/MAPK pathway, disrupting the asymmetric localization of POP-1 between the daughter cells [10]. Therefore, these cell-cycle regulators have distinct roles in the asymmetric divisions of Z1/Z4 and Z1.a/Z4.p.

Extra DTCs were also reported in *cki-1(RNAi)* animals [8]. However, the extra DTC phenotype of *cki-1* animals is different from that in *cye-1* mutants or *cdk-2(RNAi)* animals. In *cki-1(RNAi)* animals, the extra divisions are always associated with the production of extra DTCs from the Z1.a/Z4.p lineages [8]. In addition, extra DTCs can be produced by cells in the Z1.p/Z4.a lineages [8]. Such phenotypes (extra divisions and production of DTC from Z1.a/Z4.p) were not observed in *cye-1* mutants or *cdk-2(RNAi)* animals, and were suppressed in *cye-1; cki-1(RNAi)* and *cdk-2(RNAi); cki-1(RNAi)* animals. Because cyclin E is usually degraded after the G1 phase [31], one possible explanation for the extra DTCs in *cki-1(RNAi)* animals is that extra divisions of Z1.a/Z4.p progeny and possibly in Z1.p/Z4.a progeny cause the degradation of CYE-1, resulting in the derepression of the DTC fate, as occurs in *cye-1* loss-of-function mutants. In fact, in *cki-1(RNAi)* animals, we observed a small number of somatic gonadal cells that expressed a much lower level of CYE-1::GFP compared with other cells that had strong expression (data not shown). Cells with low CYE-1 activities may produce extra DTCs in *cki-1(RNAi)* animals.

Our results indicate that the balance between the levels of CYE-1 and CKI-1 determines three distinct cell states: terminal differentiation, quiescent and uncommitted, and proliferation, at least in the Z1.ap/Z4.pa cells (Fig. 4). In those cells, highly expressed *cki-1* strongly represses the low CYE-1/CDK-2 activity, blocking proliferation and permitting differentiation into DTCs. In the Z1.ap/Z4.pa cells, the low level of CKI-1 nonetheless blocks cell division by inhibiting the CYE-1/CDK-2 complex, but CYE-1/CDK-2 still represses terminal differentiation. In *cki-1(RNAi)* animals, high CYE-1/CDK-2 activities drive the cells towards proliferation. Although it remains to be determined how general this mechanism is even in *C. elegans*, our results imply that similar mechanisms may be employed in mammals to maintain cells such as stem cells in a quiescent and uncommitted state.

## Materials and Methods

### Genetic analysis

Methods for *C. elegans* culture and genetics were as described previously [35]. The *cye-1(os66)* mutants were identified in a screen for animals that lack phasmid socket cells [36]. The *os66* mutants had a nonsense mutation in the cyclin-box (W234 to stop), like the *ar95* cyclin E mutants [9]. *eh10* is a deletion mutant that lacks most of the *cye-1* coding sequence [15], [33]. *cye-1* mutants were maintained as heterozygotes over the *hT2[qIs48]* balancer. We used *qIs56* for *lag-2::GFP* [8], *maIs113* for *cki-1::GFP* [16], *maIs103* for *rnr::GFP* [16], *kuIs46* for AJM-1::GFP [27], *gvEx35* for heat-shock CYE-1 (a gift from M. Krause, NIH, Bethesda, MD) [23], *syIs49* for *zmp-1::GFP* [30], and *neEx1* for GFP::LIT-1 [18], [37]. To analyze the T-cell lineage, we observed the AJM-1::GFP expression 8 hrs after hatching, which is about 2 hours after the T.a cell division. To analyze the V6.p lineage, we observed the AJM-1::GFP expression 20 hrs after hatching. At this time, some V6.paa or V6.ppa cells (syncytial cells) had not yet fused to the syncytium (in 3/16 wild-type and 4/10 *cye-1* animals), indicating that we observed the phenotype shortly after these cells were generated.

### Analyses of CYE-1 expression

To generate the *cye-1::gfp* plasmid (pMF101), the *cye-1* genomic sequence spanning from the promoter to the region encoding the C-terminus was amplified by PCR from wild-type genomic DNA using the primers: 5′- CAGTAACCTCAAGAGTCATC-3′ and 5′-TAGGATCCGAAAAGTCGTTGCGGATG-3′. The amplified fragment was digested with *Bam*HI and ligated into the pPD95.77 vector (A gift from A. Fire), which had been digested with *Bam*HI. pMF101 was injected with *pUnc76(+)* into the *unc-76(e911)* strain as described previously [38]. The expression of GFP-fusion proteins was analyzed by confocal microscopy (LSM510 Zeiss) and fluorescence microscopy (Axioplan 2 Zeiss). The DTCs were counted after they were identified by their expression of *lag-2::GFP* and their position at the distal ends of the gonadal arms.

### Laser ablation experiments and analyses of lineages and expression

The cell ablation experiments were performed using a laser microbeam (The MicroPoint Laser System, Photonic Instruments). After ablation, the animals were recovered and grown under standard conditions. The number of DTCs was determined at the L4 or adult stage. Lineage analyses were performed according to standard methods [25].

### RNAi experiments

Fragments of exons (exon 7 for *cye-1*, exons 1 and 2 for *cki-1*, and exons 2–4 for *cdk-2*) were amplified from genomic DNA by PCR and subcloned into pGEM-T Easy (Promega). The double-stranded RNAs were then produced from the subclones by in vitro transcription with the T7 and SP6 RNA polymerases. The animals were given a dsRNA injection and grown for 12–18 hrs. Their progeny were then collected for analyses. It is reported that *cye-1(RNAi)* results in nearly complete embryonic lethality [9], [33]. However, under our conditions, the embryonic lethality was only 40% (n = 62), which allowed us to analyze the postembryonic phenotypes.
